# Spermatorrhœa

**Published:** 1859-05

**Authors:** T. A. Means

**Affiliations:** Memphis, Tennessee


ATLANTA

Medical and Surgical Journal.

VOL. IV.] MAY, 1859. [NO. IX.

### ORIGINAL COMMUNICATIONS.

### ARTICLE I.

*Spermatorrhœa.*—A Report by T. A. MEANS, M. D., of Memphis, Tennessee; read before the Medical Association of that City.

With men the genital organs are the seat of many disorders both functional and organic, a knowledge of which forms a basis for a more correct diagnosis of those disorders and of many other diseases of the organic apparatus.

Ulcerations of the penis; flow from the urethra; congenital malformation; tumors of the spermatic cord, and scrotum; cancer, hydrocele, &c., &c., are characterized as local disorders, and a diagnosis of them is generally easy.

This is not the case with certain functional derangements which result from cachexia, or some organic disease. Satyriasis, impotency, and spermatorrhœa are of this class, the latter of which shall be the subject of the present report.

Early in the month of June last, a gentleman, aged 23, came to consult me in regard to a disease which he pronounced chronic gleet, and which had been a constant source of annoyance to him, the past five years—resisting all treatment, both patent and rational.

I immediately commenced questioning him, respecting his parentage, and ancestry, going as far back as his memory would serve him, thinking I might discover the existence of some syphilitic taint, as my suspicions led me to believe, that it existed in him, and that his disease was nothing more than blennorrhagia. By a few additional questions, however, I was satisfied as to the nature of his malady, but at the same time, scrupulously avoided any question that would lead him to suspect my conviction of his having been guilty of so foul a habit as masturbation.

Growing weary with remote interrogatories, I made bold my purpose, and pressed my questions, all of which were met evasively, and so ingeniously did he fill his answers with expletives, that I almost despaired of gaining directly, the point I sought. Eventually, I pronounced that he had been guilty of onanism, and it was useless for him to conceal it longer. Yielding to the force of a professional decision so positively expressed, he acknowledged having practiced the habit, and nothing but shame and the fear of being scoffed at, had induced him to conceal it.

I elicited from him the following facts:

"In the spring of 1851—then 14 years of age—and while he was employed in a printing office, his leisure hours were taken up in listening to the idle talk among the office hands, and reading, when he could obtain them, works of a lascivious nature; women, coitus and self-pollution were fully discussed each day; the pleasures to be derived therefrom, and the expense to be incurred, &c. Having never had intercourse with women, these ideas were to him fresh and inviting. Tortured by a boyish passion, and the promptings of a secret curiosity to test the pleasures so temptingly sketched by his lewd associates, he was induced to pollute himself. Captivated by such an excess of sexual delight, he increased the practice to a habit—committing the act as often as three or four times daily, for six or eight months—then, on an average of once per week up to 1st December, '54, three years from the hour he contracted the habit, when he discovered, for the first time, an escape of semen, while at, and immediately after defecation. Emissions soon became frequent and copious, occurring during the day, and at night. Frightened by such an unusual state of things, he determined to conquer his propensity, but after one month's continence, he relapsed.

Having now the burden of the office upon his shoulders, and his disorder upon his mind, he became by degrees testy and capricious; passed, without any apparent cause, from extravagant gaiety to the most obstinate melancholy; grew petulant when advised, or spoken to; frowned at the recital of any pleasing event; shunned the society of man; loathed his occupation, and nursed with licentious zest, the habit that was consuming him.

The discharge increased in quantity and frequency, exacerbated by the least exposure, or irregularities in diet.—Immoderate sexual desire provoked a return, when the organ was passive, and not unfrequently did he experience copious discharges while quietly pursuing his daily labors, that of type setting.

His mind was less active, and his proofs but a succession of errors. Oppressed by continued ennui, and the thoughts of being turned out of employment, he was induced to make known his condition to a "quasi" druggist, who assured him, without a question, his disease was nothing more than gleet—resulting from excessive venery—and that by placing himself under his care, he would relieve him speedily, and permanently.

Flattered by such assurances, and the hope of so soon gaining his strength and spirits, gave himself up to the quack, and his drugs: tinct. iron, combined with Marshe's cordial, buttermilk, and sassafras tea were prescribed as internal remedies, with cold ablutions to the anus, perinæum, glans penis and scrotum, "pro re nata," these were advised to be kept up until he was relieved.

After following this course of treatment for six months, alternating with the bark tea and Marshe's nostrum, he perceived that the emissions became less frequent, recurring once in six days, then only in small quantities. Pleased with such a decided change, he threw up his situation, cast aside all medicine, and gave himself up to his degrading passions.

Having regained in part, his accustomed health, and animal vigor, and convinced that intercourse would further promote his recovery, yielded to the gratification with an unbridled passion, committing the act daily, and often twice, before satisfied. The effect began soon to manifest itself by weakness in the loins, with intense pains in the region of the kidneys and prostate gland.

During the third night of the second week thus occupied, he had two erotic dreams, the latter awakening him just in time to prevent an emission; which, to use his own language, he "choked" by grasping the gland firmly between his finger and thumb, so as to retain the semen within the canal, which he affirmed would so coat the mucus follicles of the urethra, that a return ot the disease would scarcely be possible. The moment, however, the organ was seized, and the emission arrested, he experienced a keen lancinating pain some inch below the glans penis, and extending itself to the prostatic region; immediately thereafter, the organ became passive, and a desire to void urine excessive.

Micturition was not difficult, nor was the flow immoderate; semen, mixed with blood, followed the last drop of urine, and a flocculent deposit was left in the chamber, resembling a thin solution of gum.

Suspecting a rupture of some blood vessel, he immediately made known the fact to his quasi physician, who, to arrest, what he termed "venous coagulated hemorrhage," gave him a solution of nitrate of silver, grs. xxv to the ℥ of water, directing him to inject, and repeat every twenty-four hours. Experiencing no relief, and growing manifestly worse, with an increased flow from the prostate, and dull and periodic pains in the region of the bladder and kidneys, he became much alarmed, and consulted another physician, who, when he approached him, pronounced his disease nothing more than blennorrhagia, with syphilitic virus, notwithstanding his positive denial of having had intercourse with woman, before the appearance of the disease.

He prescribed independently of tonics, astringent injections, acitate of lead, sulphate of copper, &c., &c., frictions with mercury, mercurial pills, hot hip baths, pedilievia, and a host of other "et cetera" without the least discretion.

His health now became more and more disordered, violent headaches constahtly annoyed him, attended with pains in the joints and loins, dizziness, inability to sleep, and great nervous excitability. Circumstances made it necessary that he should exchange a village for a city life, which was done without sacrificing his interest. Care of himself became a kind of monomania ; his strength gradually diminished, digestion was painful, imperfect and laborious. He was constantly annoyed by constipation, passing often five days withont an evacuation, to remedy which he carried in his pocket a four ounce vial of Castor Oil, taking a teaspoonful every morning and wore a woolen amulet dusted with sulphur to ward off the nightmare and regulate the secretions.

Convinced that the profession was in error, and his condition was beyond their ken, he resolved on testing his own medical skill, trusting in part to the "ipse-dixit" of Tom, Richard, and Henry, and the reported cures from the use of Holland Bitters, Iced Cordials, Brandy and Scheidam Schnapps. These proving inefficacious he concluded to test what virtue there was in a receipe handed him by a Urine Doctor, consisting of Pine and Dogwood barks made into tea—a tumblerfull three times a day with ten drops Tincture of Iron. Following this up for some two months he experienced a change for the better—the emissions recuring once in eight days, and then while asleep and in small quantities. Embolden by such a change he continued, irrregularly, the same treatment up to May '57, twenty months from date of the first dose, when, to his surprise, the discharge suddenly increased in frequency and amount, occuring at night when at stool and while the organ was passive ; soon energetic erections with veneral desire came on, causing intense pains after each discharge, the whole of which did not pass off, but remained just at the orifice of the urethra in a white bristly mass, assimilating a thick solution of Gum Arabic.

This new type in the disease excited fears of a speedy dissolution ; thoughts of suicide occupied his mind, and fancy made hideous every friendly face that looked upon him. Meeting with an experienced physician he made known his condition, history, and previous treatment. The physician believing his abstinence from woman a mere fabrication, treated him for Blenorrhagia, as others had done, directing as a preparatory course antiphlogistic remedies, i. e.: iced milk, soda and lime water, emollient injections, vegetable diet and moderate exercise. Perceiving him no better after three months treatment, he resolved upon injections with M. Lallemand's "Caustic Injector," using Nitrate of Silver in solution—ten grains to the ounce of rose water—taking internally one-fourth of a grain of Pulv. Iron on a bit of loaf sugar once in 24 hours. Injections were repeated every four days—twenty minutes to each—throughout the entire length of the urethra.

The beneficial effect of the cauterization, in conjunction with the iron, manifested itself towards the close of the second week; the discharge ceased, his features brightened and hope again returned. He could apply himself more constantly to his business, and with less fatigue than at any period since the attack—1st Dec. '54.

His skin became clear and smooth, and the cleanliness of his person, an object of his care, which before had been neglected.

His physician believing him cured enjoined upon him the necessity of marriage, as the only safeguard against future attacks to which he would be constantly liable, especially if left to satisfy his passion from the common herd.

Confiding in the knowledge and advice of his physician he married 12th Oct. '57. Being now free from restraint, physic, and disease, he commenced life anew, and left for Galveston, Texas, in the latter part of the same month, with the threefold purpose of restoring his physical man, seeing the world, and amassing a fortune. The bright dreams and flattering hopes which his condition gave, lasted but a brief season. Being a married man, and his wife his own, he felt privileged and safe in satisfying his amorous nature at pleasure; sexual indulgence became frequent, again running into excess. Towards the fourth week of marriage he discovered a slight discharge from the urethra, which increased and came on when at rest and free from excitement. His animal nature became so strong that his will could scarce control it; and violent erections would take place whenever his wife made her appearance, while mere thoughts of her did not affect him.

This sudden and unexpected return of the disease brought back the suffering of both body and mind from which he had but recently escaped. Believing his wife the cause, he concluded to send her back to reside with her parents, while he returned to the care of his malady and the judicious use of physic.

Leaving Galveston on the 23rd of April, '58, he reached Memphis on the 28th of the same month, and came to consult me on the second day after his arrival.

I found him presenting the following conditions, viz.: good stature, full chest, black hair and whiskers, florid complexion, a well developed muscular system, and to all appearances, in the enjoyment of excellent health. His look was timid and suspicious; great moral prostration; eyes languid and inexpressive; could not talk with freedom; was nervous and uneasy; seemed to flush at every question put to him; denied having had intercourse with woman, and attributed his gleet, as he termed it, to heavy lifts and irregular hours.

Upon examination, I found the genitals healthy, and his body free from eruptions or pain. He complained that vertigo, inattention, and a constant disposition to roam, harrassed him. He could not think, consecutively, twenty minutes; sleep was limited and unrefreshing; digestion imperfect and irregular; appetite good; thirst moderate; pulse free, and skin exempt from unnatural moisture.

Being unable to diagnose his disease, as blennorrhagia,

I insisted, as above said, that masturbation was the cause, spermatorhœa the effect, and that remedies were few, his condition unsafe, and cauterization the last alternative.—His history given me in the outset, led me to suspect some lesion of the urethra, resulting in stricture or cicatrix. Upon the introduction of a bougie, however, I found no obstruction; yet the patient complained of acute pain when the instrument reached the bulbous portion of the canal, but ceased the moment the bougie was withdrawn.

Being still impressed with the belief that tonics would benefit him, he insisted that I should give him some preparation of iron. To satisfy him, I prescribed, ferri subcarbonatis, ℨss; morph. sulph. grs. iv; aqua oj, directing a tablespoonful three times daily, stating at the time, I had but little confidence in the preparation.

I asked him to watch each emission closely, the quantity, color and consistence, time of occurrence, whether at night or during the day; and whether the organ was in an erect or passive state. At the close of the second week, he returned unimproved, and considerably excited, stating that the affair was growing desperate with him; and that he was willing to subject himself to any treatment that would, if not cure, at least benefit him.

I found he had experienced four pollutions, two of which occurred while in the act of defecation, one while dreaming, and a fourth, unperceived. I now put him upon a fluid regimen, consisting principally of iced milk, barley water, and simple teas, strictly forbidding the use of malt liquors of any kind; ordered warm baths, temperature 90° Far., every second morning, immediately upon rising from bed, and gentle exercise and regular hours.

This preparatory course of treatment was kept up for the space of two weeks, and with seeming benefit. His mind became more tranquil, passions less energetic, and digestion perfect.

The 11th June, I proposed cauterization, but when the day arrived, I found him unwilling to submit to the operation; his whole physique indicated a speedy dissolution; thoughts of his condition, and the situation of his wife, rendered him restless and full of sighs. Questioning him more directly as to her condition, and the cause of this sudden and unexpected depression, he frankly explained—revealing to me the fear he had long concealed, that his wife would give birth to a child before her time, and his offspring, if alive, would be idiotic, scrofulous, and full of eruptions. I arrested his fears, by assuring him all would be well; and at the same time, asked, that he would post me as to the result of her case.

Being now composed and satisfied, I mentioned, and urged upon him, cauterization. The day, and the hour arrived, but his courage again failed him; he offered sundry excuses, and begged I would allow him one week additional, for consideration. To this I gave my consent, advising him to observe the injunctions laid down, and not to void his urine for at least ten or twelve hours before reaching my office.

I met the patient on the morning of Wednesday, 21st July, and the operation was performed in the following manner—as advised by M. Lallemand, of Montpellier—the patient lying on his back, and his pelvis elevated:

I first introduced a catheter, of ordinary size, for the double purpose of drawing his urine, and to ascertain the length of the canal, position and condition of the neck of the bladder. When reaching the prostatic portion of the urethra, he manifested some uneasiness; complained of great tenderness, which increased when the instrument approached the opening of the ducts, and when reaching the bladder. Having ascertained the point I designed cauterizing, the space between the neck of the bladder and opening of the ejaculatory ducts, I withdrew the catheter and marked the length upon the "*Porte Caustique*," which I now carefully introduced, until its olivary process reached the neck of the bladder. I held the body of the instrument firmly, and adjusted the gauge so as to cauterize 1-8 of an inch of surface —forcing down the stem until exposed, I turned it briskly between my finger and thumb, and withdrew it immediately. The *whole* operation, from the introduction of a bougie to the moment of cauterization, required but ten minutes, and the *latter* but an instant.

I ordered him to remain within doors for the space of four days; diet himself strictly; use the hip-bath, once daily, at a temperature of 60°, enemeta, same temperature; to avoid extremes of heat and cold; all excesses, and keep his mind as far as possible, from his disease, and not suffer the pain and issue of blood in micturating, a result he might expect, to trouble his mind, least it might injure his condition.

I saw the patient (Mr. R.) sixteen days afterwards; he was much improved; stated he continued to void blood with his urine, until the fifth day, accompanied with pains similar to those described in gonorrhœa, but which passed off immediately. I requested that he should call to see me once a week; prescribed spiritus nitri dulcis, ʒij; balsam copaiba, ʒj; camphor mixture, ℥iv, in doses of a table spoonful morning, noon and night, keeping up a strict regimen.

Deriving some benefit from this, he called and insisted on a second cauterization, stating it was the only thing that had benefitted him, and he had made up his mind to test it fully, let the issue be good or evil. I refused him, then, as all the inflammatory symptoms set up by the first cauterization had not fully subsided. I suspended all medicine, kept him upon strict vegetable diet, cold ablutions morning and night prior to a second cauterization, which was performed 15th September, eight weeks from the first application.

I saw my patient eight days after; he seemed cheerful and content, conversed with more than his ordinary freedom, and asked the privilege to have his wife with him. I consented upon condition that he would restrict himself to moderate sexual indulgence. Emissions, other than natural, had entirely ceased, and those recurring once in ten days, then only in small quantities and without his knowledge.

On the 6th August, he came to announce the glad intelligence of his wife's safe delivery; that the condition of both mother and child was flattering, and that he had written for and expected her with him 7th September, requesting at the same time I should be specific in affixing limits to his marital intercourse, believing proper restriction impossible if left to himself. Cheered by his improved condition, and the hope of meeting his wife, he yielded to the tide of pleasure, and was again visited by the smiles which had so long forsaken him.

I saw him regularly every eight days ; improvement was manifest and progressive ; his skin clear aud fresh, and his entire form announcing health and vigor. He complained, however, of pains incomplete and fleeting in the perineum, and immoderate itching in the neighborhood of the anus, with constipation and drowsiness.

In the outset, I had directed my questions especially to the rectum, but could detect nothing that led me to suspect the existence of ascarides, until he mentioned this unpleasant sensation around the margin of the anus. I ordered douches, first very warm, then very cold, upon the perineum and loins, withholding the application when the extremes of either were felt. He experienced much relief after each bath, his skin remaining soft and cool for several hours. He kept this up for some days, when all the unpleasant symptoms vanished.

Remaining thus between improvement and cure, with the presence of his wife to cheer him, he left off all medication save that furnished by domestic affection, and found in the bosom of his home. On Saturday, 30th October, he came again with the same downcast look he had worn so impatiently the past six years, begging I should again apply the caustic, and stating that the freedom of his houshold had led him into excess, and that his emissions had again returned, and were active but scanty. Yielding to his wish, I placed him upon the same preparatory course, as on previous occasions, and on the 4th of November, I applied the caustic for the third time, with greater caution than the two preceding, and feeling satisfied that a fourth would be unnecessary.

I enjoined upon him strict abstinence from coitus for weeks, threatening if he did not withhold I would throw aside his case as hopeless, and hereafter refuse him my counsel and aid. Thus threatened into measures of safety, he nobly curbed his passion until a day specified, when I saw him.

He met me smiling, recited the past; said the present was a satisfaction, and the future was opening up a prospect bright and captivating. I discharged him 2d December, and saw no more of Mr. R. until latter part of Jan. '59. I found his condition better than at any former period; yet he still complained of immoderate itching in the neighborhood ot the anus—all other symptoms of his disease had left him, and his general health was good. I ordered a brisk enema of salt, one handfull to the pint of water, and saw the patient three days after, he entered my office, pale, irresolute, and manifestly nervous; said every time he made water, blood came with his urine, and that the injection was so strong, he could not hold it in the canal as long as I directed. Vexed almost to madness, at such an unpardonable blunder, and yet red with laughter, I asked if he had injected the strong brine up the *urethra?* He said he had, and that blood came immediately after. Pushing my questions further, I found he had forced the nozzle of a pt. syringe, full an inch and a half up the urethra, excoriating its surface, so that, the moment the solution came in contact with the part abraded, the pain came intense; excited his fears, and brought back the inquisitive looks and broken sighs that the past used to exhibit.

Sympathizing with, and assuring him, that no serious evil would result, I bade him adieu, with the request, should a return, or symptoms of a return, manifest themselves, that he would inform me. With this promise, he left me, since which time, I have not seen him, and therefore reasonably infer, the cure to be complete.

In the case just reported, nothing of a peculiar or startling nature presents itself, yet, the class of diseases to which it belongs, are of a more serious nature than the profession generally suppose; and this, among the number, has been sadly overlooked. It is doubtless the germ from which springs almost all diseases under the head of non-syphilitic blennorrhagia, gleet, impotency, &c. Congenital predisposition and debility; hereditary transmission; sickly childhood; phimosis and paraphimosis; strains, falls, or blows; excessive use of spirituous or malt liquors; irritation kept up by ascarides; abuse of strong medicines, cantharides, camphor, &c.; horseback exercise; influences of the brain; weak, and naturally delicate generative organs; highly imaginative mind; inordinate venereal excitement; sleeping under too much cover; and a too constant craving after the opposite sex, without gratification; protracted constipation; and undue excitement of the genitals during infancy; warm latitudes, and vitiated habits, are prominent in provoking this disease, and may be termed primary or predisposing causes, while titillating the genital organs may be considered the immediate or mechanical cause.

At the age of puberty, when the passions of youth are fresh, and ardent thoughts of coitus are allowed constantly to occupy the mind, and keep up a continual warfare with the judicious admonition and counsel, received from an anxious and devoted parent—thus contending between parental discipline and the promptings of immoderate sexual desires, the young sensualist seeks to gratify himself by mechanical means, and aloof from mankind. Once addicted, he looks no longer to woman to meet his amorous wants, but cloistered and alone, measures his present joys without estimating his future ills.

In almost every protracted case of gleet, following blennorrhagia, do we find more or less inflammation of the prostate, and a general debilitated condition of the seminal ducts which aggravates the disease, and render it intractible and provoking. The organs, too, of secretion and excretion, are diseased, although our books teach us that the discharge is of an inflammatory nature, and confined alone to the urethra in the male, and the vagina in the female, and does not involve directly any neighboring part, yet, by neglect, or excess of any kind, a disease may be produced that will outlive all medication.

Question a man who has a flow from the urethra follow ing a protracted case of Blemorrhagia, and he will tell you that in early life he had practiced the secret vice, and that he had successfully concealed the fact and indured the torture, both of body and mind, with the fortitude of a martyr, fearing lest an acknowledgement would expose him, prostrate his hopes and ruin his honor. Thus secretly does he wear a habit so progressive and dangerous, and one too that sooner or later must work a change that will resist all treatment, and leave the organs of excretion so enervated, that the most moderate sexual indulgence will produce what is termed non-syphilitic Blemorrhagia.

The organs thus whipped into action by no other than mechanical exercise, must leave them weak and diseased. Thus it is we hear the cry of Gonorrhœa from married men, who had but an hour before implicit confidence in the chastity of their wives—denouncing them as vile and diseased. Sound them thoroughly as before said, and you will find this secret vice—practiced in childhood and adult age—to be the ground work of their malady and distrust—their wives free from disease, and their imputation as ungenerous as unjust. Generally in all such cases no treatment but abstinence and care should be instituted or advised, as it will run its course in 16 or 20 days, aided by simple injections of Port Wine after second week of attack.

In the general appearance of the patient, mentioned in the report just read, nothing could have been detected by a casual observer that would lead him to suspect the existence of disease, yet when examined and closely questioned, symptom after symptom appeared which confirmed his malady. His history once satisfactorily obtained, and his disease established we have half the remedy, and by a judicious and very limited use of medicine cure may be effected. Astringent injections, cubebs and balsams should be seldom administered and then only to satisfy the whim or caprice of your patient. The only remedy I have yet found to perfect a cure is cauterization as advised and practiced by M. Lallemand, and to save further remarks I would refer for detail and treatment to that author's excellent work on Spermatorrhœa.

Diagnosis of this disease is easy and may be readily established by asking the patient two simple questions: At what time does the emission occur? Before or after micturition? If he should answer *before*, pronounce it Blennorhagia, if *immediately following* the last drop of urine, call it Spermatorrhœa, and nothing else.

Gonorrhœa Pura, or non-syphilitic Blennorrhagia is only distinguished from Spermatorrhœa by the absence of the discharge after passing urine. Gonorrhœa Impura results from impure commerce. Gonorrhœa Pura does not follow impure connection, the one is characterized by a mucus discharge from the urethra intermixed with specific matter, the other by no great local inflammation, less discharge, greater pain *after*, and less *during* micturition, while the flow in Spermatorrhœa takes place periodically without pain and always AFTER voiding urine.

